# Supplementary material for: Nature-inspired hierarchical building materials with low CO2 emission and superior performance
Source: Nat Commun. 2025 Mar 28;16:3018. doi: 10.1038/s41467-025-58339-8 (PMC11950366; doi:10.1038/s41467-025-58339-8)
Supplement: Supplementary file 1 — Supplementary Information [file 41467_2025_58339_MOESM1_ESM.pdf]

Supplementary Information

Nature-Inspired Hierarchical Building Materials with Low CO_2 Emission and Superior Performance

Jinyang Jiang[a], Han Wang[a], Junlin Lin[a], Fengjuan Wang[a], Zhiyong Liu[a], Liguo Wang[a], Zongjin Li[b], Yali Li[c], Yunjian Li[b*], Zeyu Lu[a*]

a. Jiangsu Key Laboratory of Construction Materials, School of Materials Science and Engineering, Southeast University, Nanjing 211189, China;
b. Centre for Smart Infrastructure and Digital Construction, School of Engineering, Swinburne University of Technology, Hawthorn, Victoria, 3122 Australia.
c. Faculty of Innovation Engineering, Macau University of Science and Technology, Macau SAR, 999078, China

Corresponding Author[*]: Yunjian Li, *liyunjian@must.edu.mo*; Zeyu Lu, *101012819@seu.edu.cn.*

1. Microstructure

S.1 (a-b) indicated the shape of pores in LLST-1 was similar with that in hydrogel, due to the template role of hydrogel. In contrast, as depicted in S.1 (c), the pores in Ref. exhibited irregular shapes, leading to stress concentrations under loading. During the hydration process (S. 1 (d-f)), the micropores in hydrogel skeleton were filled by uniform dispersed cement hydrates containing nanopores, leading to the construction of micro/nano-hierarchical porous structure.

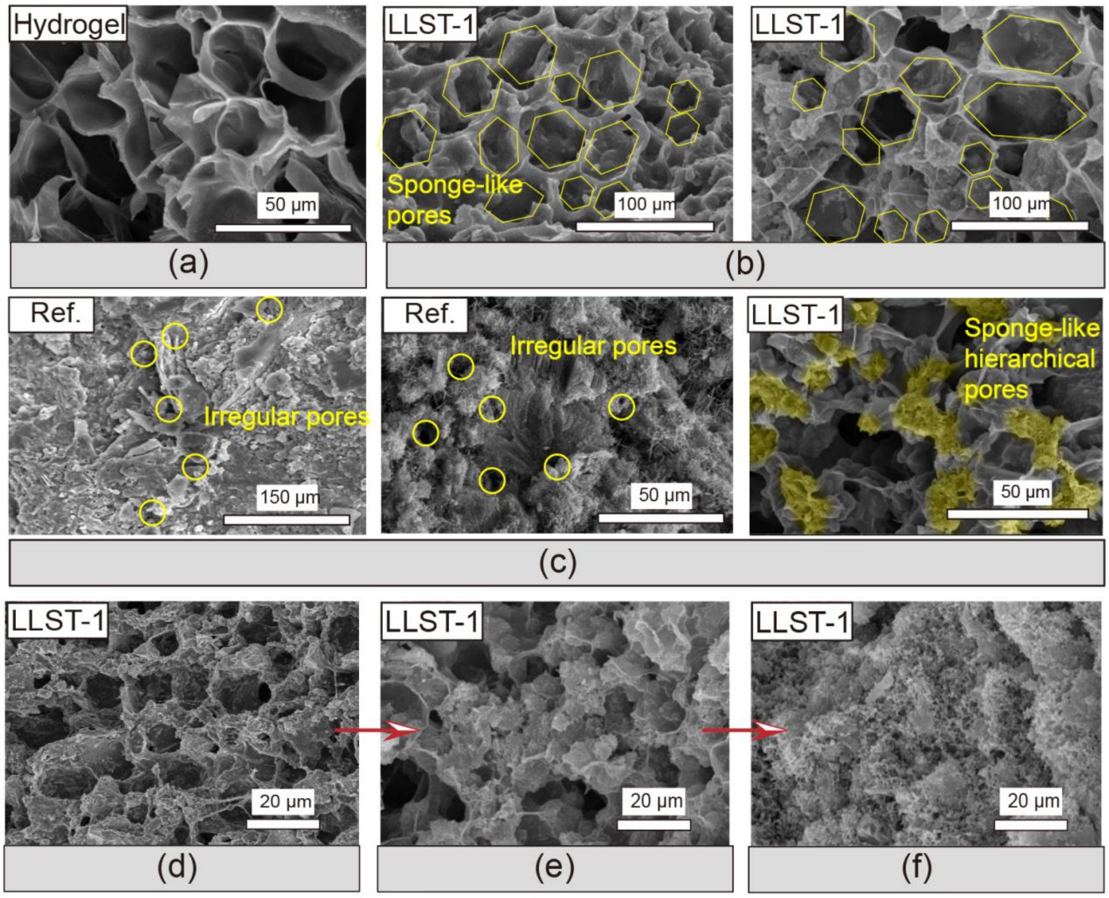

S. 1. Microstructure of pure hydrogel and LLST at different hydration ages. (a) Pure hydrogel. (b-c) Sponge-like pores in LLST. (c) Pore shape in Ref. (irregular) and LLST (Sponge-like hierarchical pores). (d-f) Microstructure of LLST after 10 min of fabrication, 7 and 28 days of hydration, respectively.

2. Pore structure

As shown in S.2 (a), the total porosity of LLST ranged from 36.9% to 75.2%, significantly higher than that of Ref. This finding aligned with the MIP results. Additionally, as shown in S.2 (b), the density of LLST ranged from 0.75 to 1.45 g/cm^3, which was 12% to 54% lower than that of Ref. Notably, the porous structure endowed LLST with low thermal conductivity (0.18 W/m·K), as shown in S.2 (c), which is 85% lower than that of normal cement paste[1]. Consequently, LLST exhibited great potential for applications in thermal insulation materials.

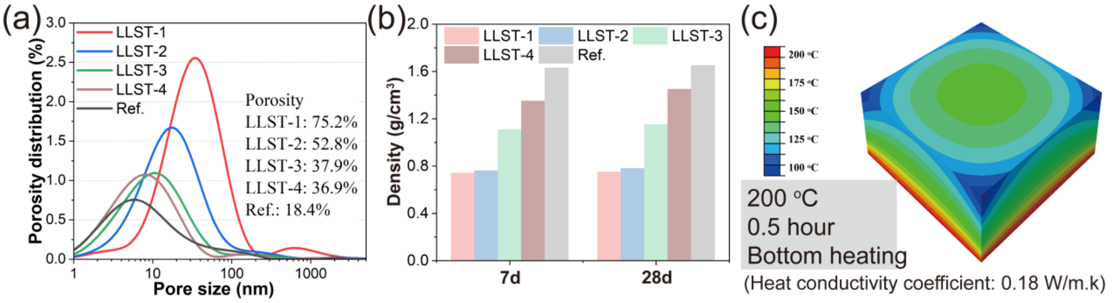

S. 2. Porosity, density and thermal conductivity of LLST. (a) Pore size distribution and total porosity of LLST. (b) Density of LLST. (c), Simulation of insulation performance.

3. Strength

As shown in S.3 (a), the flexural strength, maximum deformation rate, and fracture energy of LLST were 102% to 1365% higher than those of Ref. Additionally, S.3 (b) illustrated that Ref. exhibited a quasi-brittle failure mode, where stress increased rapidly with strain until reaching the yield point, followed by a sudden drop in stress. The fracture toughness of LLST, as presented in S-Table 1, was 44% to 120% higher than that of Ref. Furthermore, S-Table 2 provides a comparison of density and compressive strength data for foam cement from the literature. The results demonstrated that LLST achieved a lower density while exhibiting superior mechanical strength compared to foam cement.

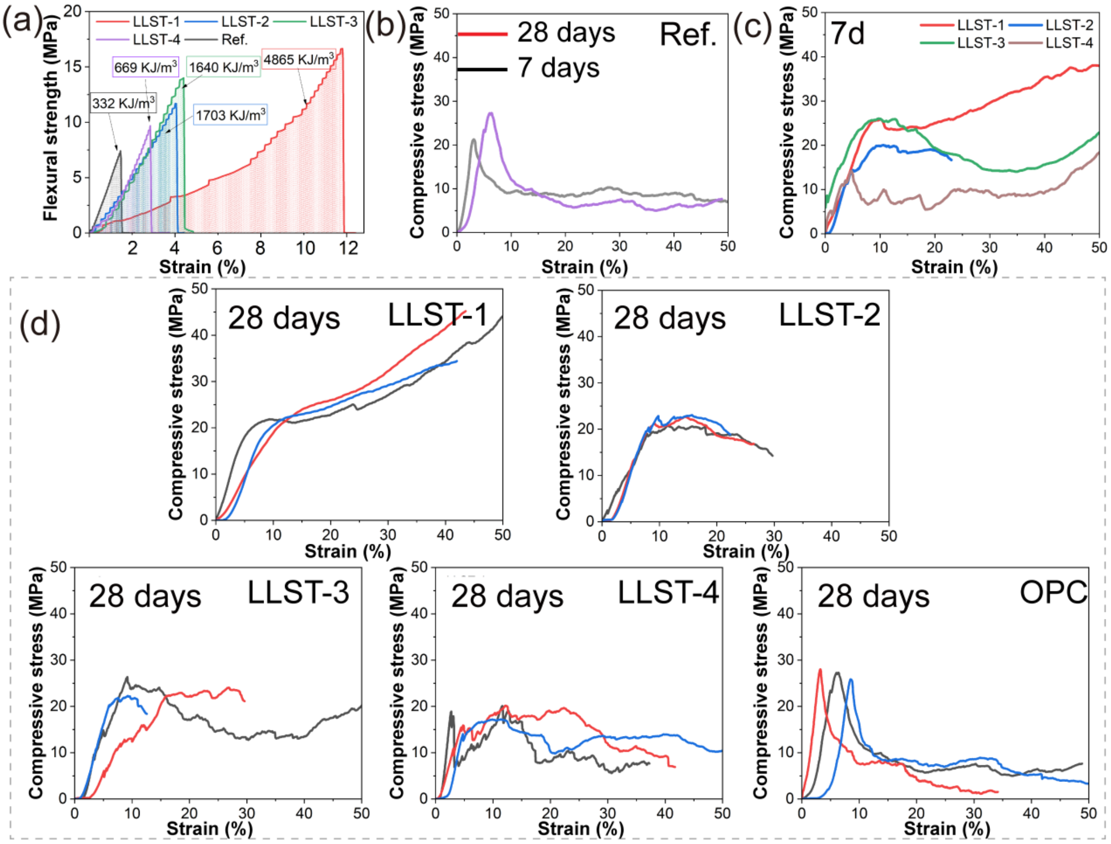

S. 3. Mechanical strength of LLST. (a) Midspan deformation rate and fracture energy

of LLST at 28 days. (b-c) Load-displacement curve of Ref., and LLST. (d) Strain-compressive stress curve of LLST.

S-Table 1. Fracture toughness of LLST.

Specimen	a/W	f(a/W)	Pmax (N)	KIC ($MPa \cdot m^{1/2}$)	Average ($MPa \cdot m^{1/2}$)	Improvement (%)
LLST1	0.491	2.589	212.000	0.631	0.609	119.9
	0.497	2.639	204.000	0.619		
	0.486	2.549	197.000	0.577		
LLST2	0.493	2.606	194.000	0.581	0.561	102.5
	0.499	2.656	185.000	0.565		
	0.497	2.639	177.000	0.537		
LLST3	0.504	2.700	178.000	0.552	0.518	87.0
	0.496	2.631	165.000	0.499		
	0.489	2.573	170.000	0.503		
LLST4	0.495	2.623	110.000	0.331	0.398	43.7
	0.498	2.648	144.000	0.438		
	0.506	2.717	136.000	0.425		
Ref.	0.496	2.631	110.000	0.333	0.277	-
	0.498	2.648	87.000	0.265		
	0.492	2.598	78.000	0.233		

S-Table 2. Summary of foam cement properties.

Composition	w/c	Compressive strength (MPa)	Dry Density (kg/m^3)	Foaming materials type
Portland cement, Sand[2]	0.4~0.5	5.5~24.3	1300~1600	Protein-based foam agent
Portland cement, Glass powder/ Thermoplastic powder[3]	0.45~0.75	1.5~10.3	800~1580	Protein-based foam agent
Portland cement, Sand[4]	0.9~1	1.0~7.0	800~1350	Organic foaming agent
Portland cement, Sand[5]	0.9~1	2.0~11.0	650~1200	Organic foaming agent
Portland cement, Sand, Rubber[6]	0.38~0.5	6.4~18.3	1500~1660	Organic foaming agent
Portland cement[7]	0.4~0.60	0.1~6	400~800	Protein-based foam agent
Portland cement, Fly ash[8]	0.33~0.36	8.2~10.4	970~1307	Organic foaming agent
Portland cement, Slag[9]	0.52	0.4~0.8	150~300	H_2O_2
Portland cement, Sand[10]	0.4~0.6	2.0~11	650~1200	Organic foaming agent
Portland cement, Sand, Slag[11]	0.55~0.91	1.1~2.0	975~1132	Protein-based foam agent

4. Computational methods

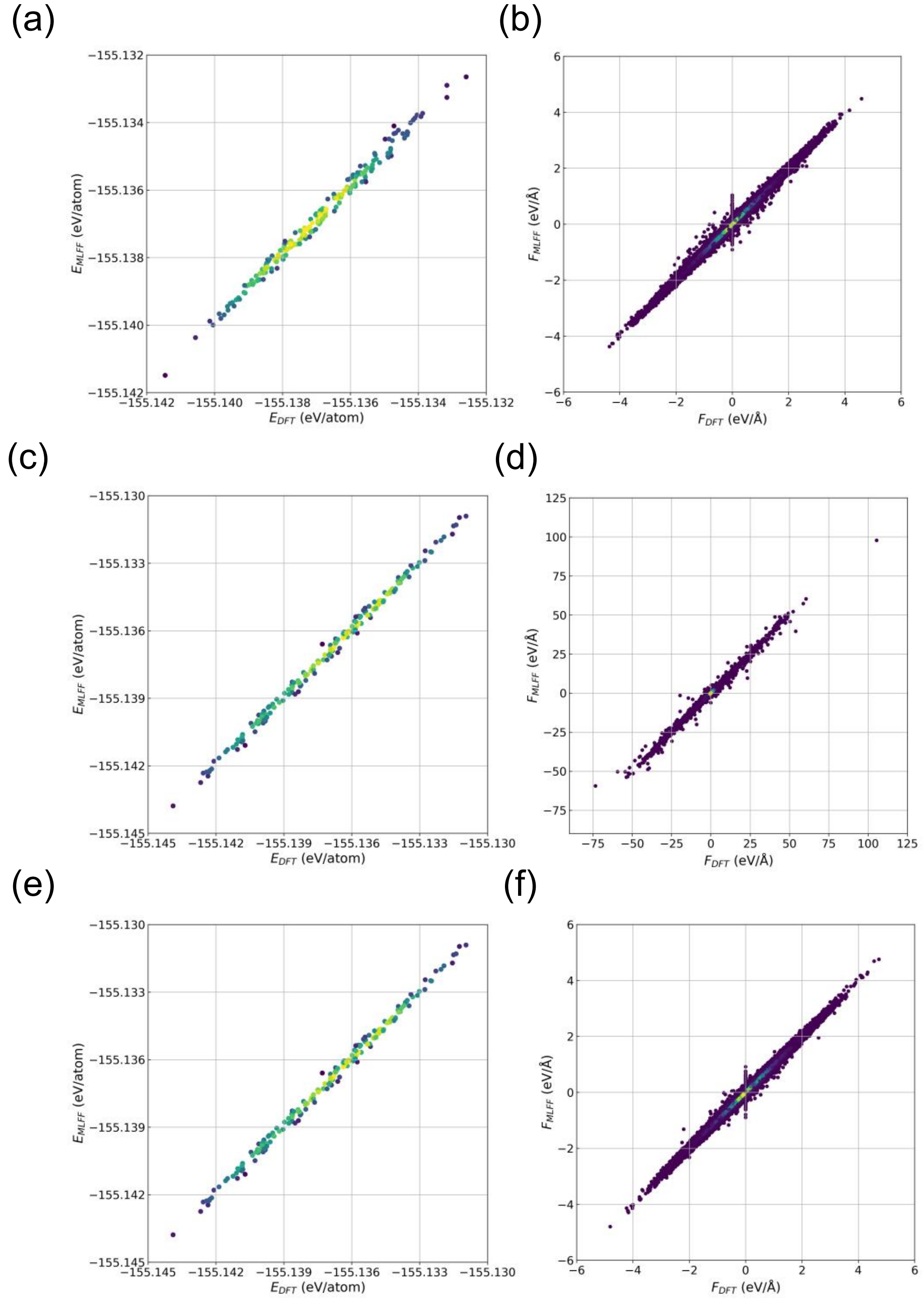

S. 4. Comparison of the energies and atomic forces calculated by the MLFF and DFT. (a) and (b) Comparison for configurations in the AIMD-based WT-MetaD training dataset. (c) and (d) Comparison for configurations in the perturbed structure training dataset. (e) and (f) Comparison for configurations in the test dataset.

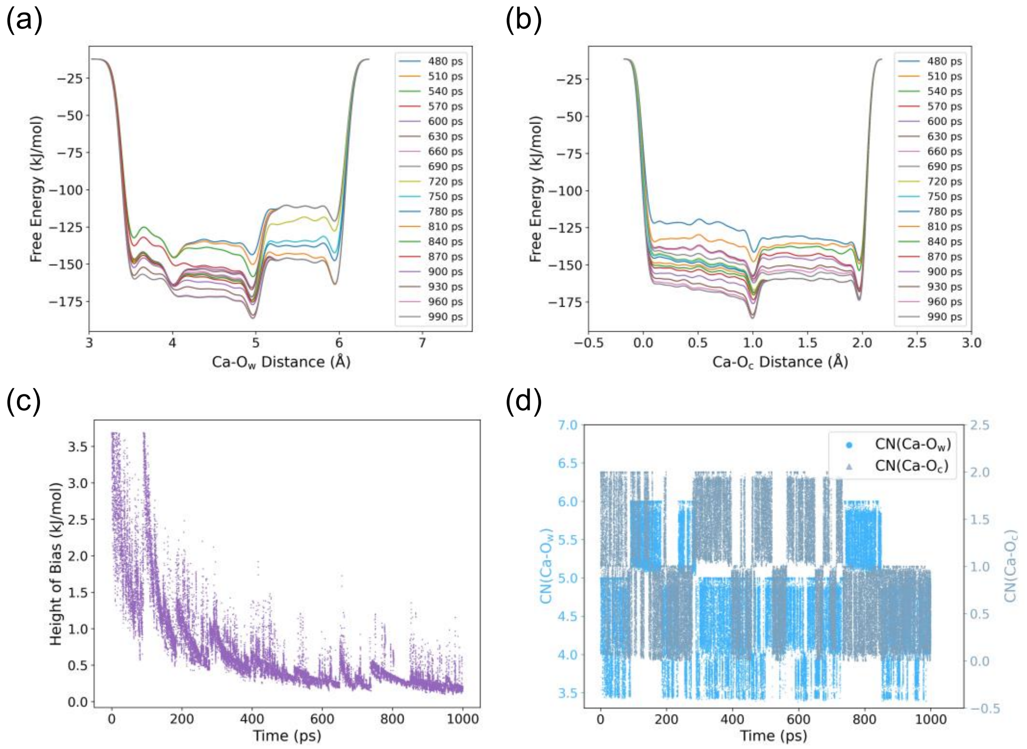

S. 5. Convergence tests for the WT-MetaD simulation with the collective variable of CN(Ca-O_w) and CN(Ca-O_c). (a) and (b) Convergence tests for free energy surfaces as a function of CN(Ca-O_w) and CN(Ca-O_c), which is performed every 30 ps (1000 Gaussian kernels deposited) along the 1 ns simulation time, the metadynamics is converged after 1 ns. (c) Time evolution of the height of the bias added to the system, the height of the bias decreases with the time. (d) Time evolution of CN(Ca-O_w) and CN(Ca-O_c) during the simulation time.

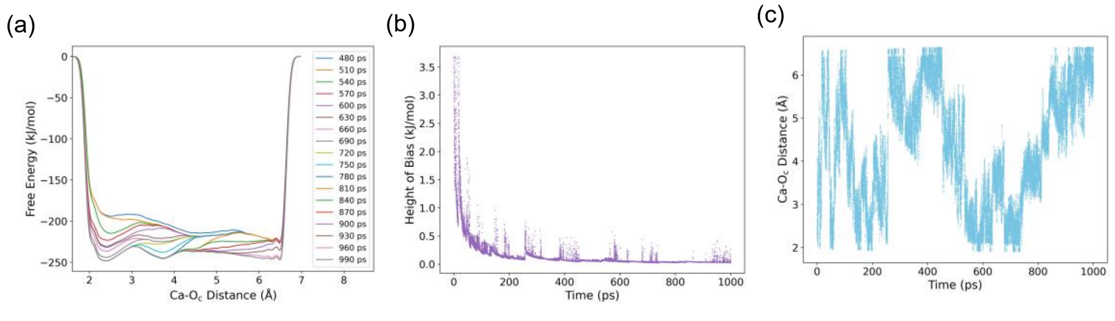

S. 6. Convergence tests for the WT-MetaD simulation with the collective variable of Ca-O_c distance. (a) Convergence tests for free energy surfaces as a function of Ca-O_c distance, which is performed every 30 ps (1000 Gaussian kernels deposited) along the 1 ns simulation time. The metadynamics is converged after 1 ns. (b) Time evolution of the height of the bias added to the system. The height of the bias decreases with the time. (c) Time evolution of Ca-O_c distance during the simulation time.

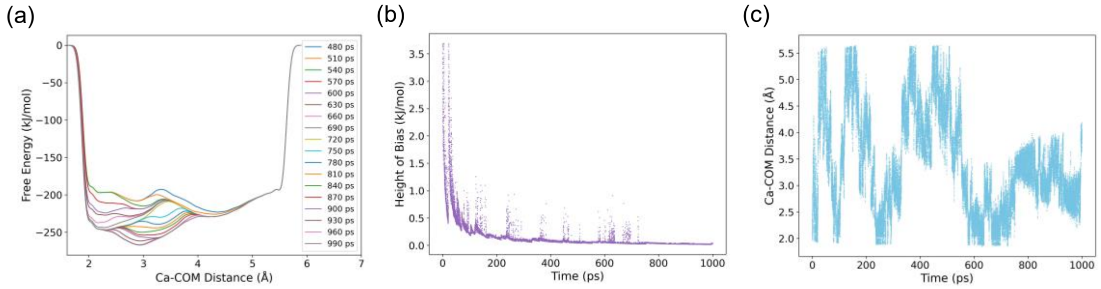

S. 7. Convergence tests for the WT-MetaD simulation with the collective variable of Ca-COM distance. (a) Convergence tests for free energy surfaces as a function of Ca-COM distance, which is performed every 30 ps (1000 Gaussian kernels deposited) along the 1 ns simulation time. The metadynamics is converged after 1 ns. (b) Time evolution of the height of the bias added to the system. The height of the bias decreases with the time. (c) Time evolution of Ca-COM distance during the simulation time.

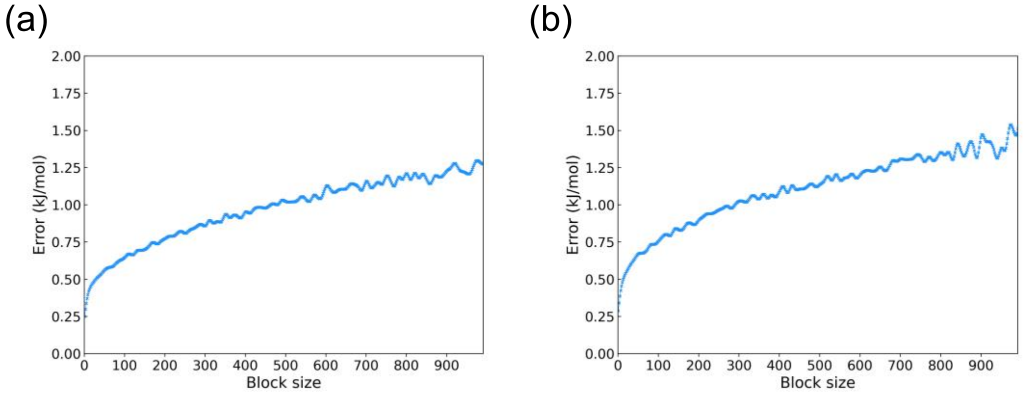

S. 8. Block analysis of (a) MLFF 2 and (b) MLFF 3 simulations for the average errors in different blocks from 0 to 1000.

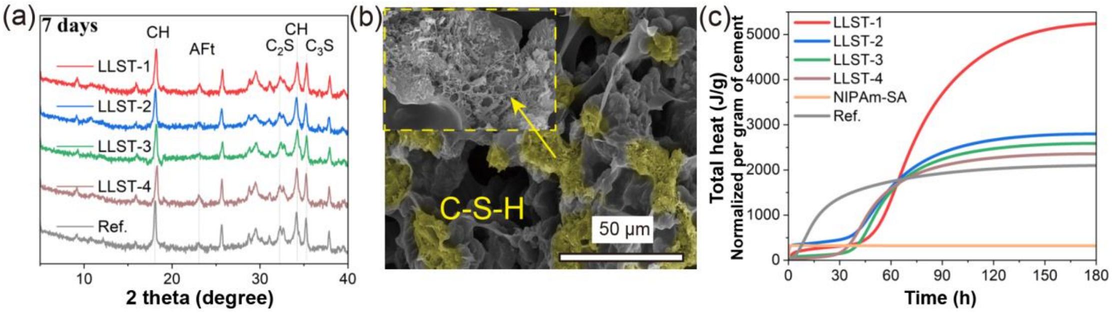

S. 9. Hydration characteristics of LLST. (a) Types of cement hydrates after 7 days of cement hydration. (b) Formation of C-S-H on hydrogel skeleton. (c) Total heat within 7 days of cement hydration.

5. Influence of cement pore solution on hydrogel gelation

The influence of cement pore solution on hydrogel gelation was investigated in S. 10. The results indicated that the gelation of hydrogel can be accomplished in cement filtrate. However, compared with DI water, the gelation rate of hydrogel was delayed. This was because the $S_2O_8^{2-}$ from APS (potassium persulfate) was consumed by OH^- in cement filtrate[12].

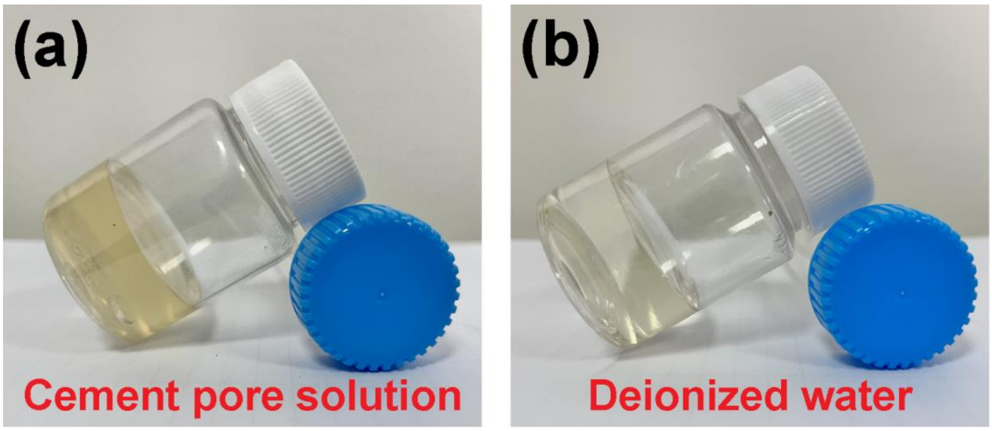

S. 10. Gelation of hydrogel in (a) cement pore solution and (b) deionized water.

6. Life cycle assessment of LLST

6.1. Scope of the study

The present study aimed to determine the environmental impact of the LLST production[13, 14]. The production of 1 cubic meter of LLST represented the functional unit of the systems considered in the LCA assessment.

6.2. System boundary for LCA

The system boundaries considered in this study included the following aspects:

The system boundaries included in this study includes the following aspects:

(1): The raw materials included Cement (P·I 42.5 type), N-isopropylacrylamide (NIPAm), sodium acrylate (SA), N,N-methylenebisacrylamide (Bis), potassium persulfate (APS), and N,N,N'N'-tetramethylethylenediamine (TEMED).

(2): The environmental impacts of raw materials production, mainly generated by the fuel consumption in the production and transportation, as well as the decomposition of raw materials during manufacturing.

(3): The environmental impact of LLST production, mainly generated by the fuel consumption in the production and transportation.

(4): Potential impact of using organic materials.

The following cases in S-Table 3. are calculated in LCA:

S-Table 3. Different cases of LCA calculation.

Mix. (ton)	LLST-1	LLST-2	LLST-3	LLST-4	Ref.
Cement	0.43	0.75	1	1.2	1.2
NIPAm	0.048	0.048	0.048	0.048	-
SA	0.012	0.012	0.012	0.012	-
Bis	0.0009	0.0009	0.0009	0.0009	-
APS	0.0009	0.0009	0.0009	0.0009	-
TEMED	0.00072	0.00072	0.00072	0.00072	-

6.3. Life cycle inventory

The global warming potential (GWP), acidification potential (AP), eutrophication potential (EP) of raw materials were considered.

It should be noted that emissions of cement included raw material decomposition, calcination fuel, transportation fuel, and electricity consumption. The emissions of organic included electricity consumption and transportation fuel.

S-Table 4. Emissions from the production of raw materials[15,16, 17, 18].

Mix. (per ton)	GWP (kg CO_2)	AP (kg SO_2)	EP (kg NO_X)
Cement	9.40 E+02	5.00 E-01	1.53 E+00
NIPAm	3.1 E+03	7.10 E+00	5.08E-1
SA	2.6 E+03	8.49 E+00	1.01 E-00

S-Table 5. Emissions from the transportation of raw materials[19, 20].

Mix. (per ton)	GWP (kg CO_2)	AP (kg SO_2)	EP (kg NO_X)
Road transportation (Diesel)	1.63 E+02	2.00 E+00	1.09 E+00

Note: the transport distance here was set as 100 km.

S-Table 6. Emissions from the production of LLST[19].

Mix (per m^3)	GWP (kg CO_2)	AP (kg SO_2)	EP (kg NO_X)
LLST products	3.05 E+00	-	-

6.4. Impact Assessment

The impact categories considered in this research included: (1) Global Warming Potential (GWP): Emissions associated with CO_2; (2) Acidification Potential (AP): Emissions that caused acid rain; (3) Eutrophication Potential (EP): Nutrients emissions leading to water body eutrophication.

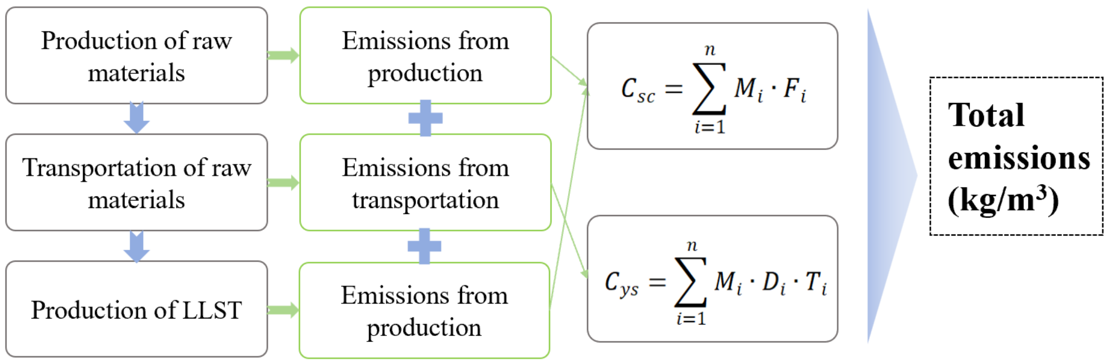

S. 11. Computing model of LCA for LLST.

The details of the LCA of LLST cement were displayed as follows, which contained four formulas:

$$C_{jc} = C_{sc} + C_{ys} \quad (1)$$

C_{jc}: Emissions during the production and transportation of each building materials; C_{sc}: Emissions from production stage of each building materials ($kgCO_2e$); C_{ys}: Emissions from Transportation stage of each building materials ($kgCO_2e$).

$$C_{sc} = \sum_{i=1}^{n} M_i \cdot F_i \quad (2)$$

M_i: Consumption of each building materials (ton); F_i: Emissions factor of LLST per unit weight (kg/ton).

Emissions from the production of LLST (F_{ip}) were also taken into F_i, according to the formula (3):

$$F_{ip} = \frac{E_{ip} \cdot k_{ip}}{N} \quad (3)$$

F_{ip}: Emissions from the production of LLST; E_{ip}: The total amount of electricity consumed; k_{ip}: Emission factor of electric energy (around 086 (kg/kWh)); N: Total amount of LLST productions.

$$C_{ys} = \sum_{i=1}^{n} M_i \cdot D_i \cdot T_i \quad (4)$$

M_i: Consumption of each raw materials (ton); D_i: Average transport distance of each raw materials (km); T_i: Emission factor of each raw materials per unit weight and per unit transportation distance ($kgCO_2e$/(ton·km)).

6.5. Interpretation of Results

S-Table 7. Emissions from the LLST.

Mix. (ton)	GWP (kg CO_2)	AP (kg SO_2)	EP (kg NO_X)
LLST-1	684.79	1.51768	1.16656
LLST-2	1037.75	2.31768	2.00496
LLST-3	1313.5	2.94268	2.65996
LLST-4	1534.1	3.44268	3.18396
Ref.	1354.1	3	3.144

Analysis of Findings: The life cycle assessment results indicated that the LLST was characterized with a significantly less amount of cement thus reducing 3% ~ 49% of GWP, 2% ~ 50% of AP and 16% ~ 63% of EP from the production and transportation process. The LCA results proved that, compared with conventional cement-based materials, the application of LLST was beneficial for the environment. In addition, it should be noted that CO_2 emissions from the production of chemical products primarily originated from energy consumption, which could be mitigated by using clean energy. However, approximately 60% of the CO_2 emissions from cement-based materials resulted from the decomposition of $CaCO_3$, which was unavoidable. These findings underscored that the newly developed LLST functioned as a low-carbon building material with significant potential to reduce carbon emissions in the cement industry.

7. Information of raw materials

S-Table 8. Phase composition of cement.

	C_3S	C_2S	C_3A	C_4AF
Cement	62.47	16.85	5.51	12.58

S-Table 9. Chemical composition of cement.

	CaO	SiO_2	Al_2O_3	SO_3	Fe_2O_3	LOI
Cement	63.88	21.31	5.67	2.32	3.74	1.53

References:

1. Chen Y., et al. Multi-layered cement-hydrogel composite with high toughness, low thermal conductivity, and self-healing capability. Nat. Commun. **14**, 3438 (2023).
2. Bagheri A., Samea S.A. Role of non-reactive powder in strength enhancement of foamed concrete. Constr. Build. Mater. **203**, 134-145 (2019).
3. Chandni T.J., Anand K.B. Utilization of recycled waste as filler in foam concrete. J. Build. Eng. **19**, 154-160 (2018).
4. Nambiar E., Ramamurthy M. Fresh State Characteristics of Foam Concrete. J. Mater. Civ. Eng. **20**, 111-117 (2008).
5. E. K. Kunhanandan Nambiar, K. Ramamurthy MA. Shrinkage Behavior of Foam Concrete. J. Mater. Civ. Eng. **21**, 631-636 (2009).
6. Eltayeb E., Ma X., Zhuge Y., Youssf O., Mills J.E. Influence of rubber particles on the properties of foam concrete. J. Build. Eng. **30**, 101217 (2020).
7. Liu Z., Zhao K., Hu C., Tang Y. Effect of Water-Cement Ratio on Pore Structure and Strength of Foam Concrete. Adv. Mater. Sci. Eng. **10**, 1-9 (2016).
8. Markin V., Nerella V.N., Schrofl C., Guseynova G., Mechtcherine V. Material Design and Performance Evaluation of Foam Concrete for Digital Fabrication. Materials **12**, 2433 (2019).
9. Pan Z., Li H., Liu W. Preparation and characterization of super low density foamed concrete from Portland cement and admixtures. Constr. Build. Mater. **72**, 256-261 (2014).
10. Ramamurthy K., Kunhanandan N., Indu S. A classification of studies on properties of foam concrete. Cem. Concr. Compos. **31**, 388-396 (2009).
11. Oren O.H., Gholampour A., Gencel O., Ozbakkaloglu T. Physical and mechanical properties of foam concretes containing granulated blast furnace slag as fine aggregate. Constr. Build. Mater. **238**, 117774 (2020).
12. Sun G., Li Z., Liang R., Weng L.T., Zhang L. Super stretchable hydrogel achieved by non-aggregated spherulites with diameters <5 nm. Nat. Commun. **7**, 12095 (2016).
13. Stafford F.N., Dias A.C., Arroja L., Labrincha J.A., Hotza D. Life cycle assessment of the production of Portland cement: a Southern Europe case study. J. Clean. Prod. **126**, 159-165 (2016).
14. Ige O.E., Olanrewaju O.A., Duffy K.J., Obiora C. A review of the effectiveness of Life Cycle Assessment for gauging environmental impacts from cement production. J. Clean. Prod. **324**, 129213 (2021).

15. Rashid A., Khan S.A., Koç M. Life cycle assessment on fabrication and characterization techniques for additively manufactured polymers and polymer composites. Clean. Environ. Syst. **12**, 100159 (2024).
16. Petrescu L., Fermeglia M., Cormos C. Life Cycle Analysis applied to acrylic acid production process with different fuels for steam generation. J. Clean. Prod. **133**, 294-303 (2016).
17. Van P., Mignon A., Habert G., De Belie N. Cradle-to-gate life cycle assessment of self-healing engineered cementitious composite with in-house developed (semi-)synthetic superabsorbent polymers. Cem. Concr. Compos. **94**, 166-180 (2018).
18. Wang Y., et al. Life cycle assessment of a novel biomass-based aerogel material for building insulation. J. Build. Eng. **44**, 102988 (2021).
19. Xu K., Kang H., Wang W., Jiang P., Li N. Carbon Emission Estimation of Assembled Composite Concrete Beams during Construction. Energies **14**, 1810 (2021).
20. Machado P.G., Teixeira A., Collaço F., Mouette D. Review of life cycle greenhouse gases, air pollutant emissions and costs of road medium and heavy-duty trucks. WIREs Energy Environ. **10**, 395 (2021).
